# Estimation of the haematological toxicity of minor groove alkylators using tests on human cord blood cells.

**DOI:** 10.1038/bjc.1997.155

**Published:** 1997

**Authors:** M. Ghielmini, G. Bosshard, L. Capolongo, M. C. Geroni, E. Pesenti, V. Torri, M. D'Incalci, F. Cavalli, C. Sessa

**Affiliations:** Servizio Onclogico Cantonale, Ospedale S. Giovanni, Switzerland.

## Abstract

We evaluated the myelotoxicity and the anti-tumor potential of tallimustine, three of its analogues and carzelesin, with melphalan as reference substance. Tallimustine was tested by clonogenic assays on both human bone marrow (BM) and cord blood (hCB) cells, the other compounds on hCB only. The degree of inhibition of the haemopoietic progenitors GM-CFC, CFC-E and BFU-E was evaluated after exposure to different concentrations. The same schedules were tested on five tumour cell lines. We found that the dose-response curves for tallimustine on BM and hCB cells were similar. Carzelesin was shown to be the most potent of the substances tested and to be the one with the best in vitro therapeutic index; of the distamycin analogues, the one bearing an alpha-bromoacrylic group (FCE 25450) had the best index. For melphalan, tallimustine and carzelesin, the concentration inhibiting the growth of 70% of progenitor cells in vitro (ID70) was similar to the concentrations found in the serum of patients treated at the maximum tolerated dose (MTD). We conclude that hCB cells may be used instead of BM cells for in vitro myelotoxicity tests. Therapeutic indexes can be extrapolated from this model and could help in selecting the most promising analogue for further clinical development. The in vitro-active concentrations are similar to myelotoxic concentrations in patients, suggesting a predictive value for the assay.


*British Journal of Cancer* (1997) **75**(6), 878–883
© 1997 Cancer Research Campaign

### Estimation of the haematological toxicity of minor groove alkylators using tests on human cord blood cells

**M Ghielmini[1], G Bosshard[1], L Capolongo[2], M C Geroni[2], E Pesenti[2], V Torri[3], M D'Incalci[3], F Cavalli[1] and C Sessa[1]**

[1]Servizio Oncologico Cantonale, Ospedale S. Giovanni, 6500 Bellinzona, Switzerland; [2]Pharmacia-Upjohn, Exp. Oncology Laboratory, Via Giovanni XXIII, 20014 Nerviano, Italy; [3]Dipartimento di Oncologia, Istituto Mario Negri, Via Eritrea 62, 20157 Milan, Italy

**Summary** We evaluated the myelotoxicity and the anti-tumor potential of tallimustine, three of its analogues and carzelesin, with melphalan as reference substance. Tallimustine was tested by clonogenic assays on both human bone marrow (BM) and cord blood (hCB) cells, the other compounds on hCB only. The degree of inhibition of the haemopoietic progenitors GM-CFC, CFC-E and BFU-E was evaluated after exposure to different concentrations. The same schedules were tested on five tumour cell lines. We found that the dose–response curves for tallimustine on BM and hCB cells were similar. Carzelesin was shown to be the most potent of the substances tested and to be the one with the best in vitro therapeutic index; of the distamycin analogues, the one bearing an alpha-bromoacrylic group (FCE 25450) had the best index. For melphalan, tallimustine and carzelesin, the concentration inhibiting the growth of 70% of progenitor cells in vitro ($ID_{70}$) was similar to the concentrations found in the serum of patients treated at the maximum tolerated dose (MTD). We conclude that hCB cells may be used instead of BM cells for in vitro myelotoxicity tests. Therapeutic indexes can be extrapolated from this model and could help in selecting the most promising analogue for further clinical development. The in vitro-active concentrations are similar to myelotoxic concentrations in patients, suggesting a predictive value for the assay.

**Keywords:** carzelesin; tallimustine; clonogenic tests; toxicology

Phase I clinical trials remain pivotal in the development of new anti-cancer drugs, but more refined methods are needed to minimize the number of patients treated with inactive dosages. Traditionally, the starting dose for phase I studies corresponds to one-tenth of the mouse $LD_{10}$, with subsequent dose escalations according to a modified Fibonacci scheme, however methods to reduce the number of dose levels have been proposed in recent years. One of them, the 'pharmacologically guided dose escalation' method, relies on the observation that for many (but not all) drugs plasma levels in mice at the $LD_{10}$ are similar to those found in humans at the MTD, and that similar AUC values in mice and humans will produce similar toxic effects (Davis et al, 1988; Collins et al, 1990; Gianni et al, 1990). Nevertheless, because of interspecies variations in schedule dependency, tissue distribution and steepness of the slope of the toxicity vs concentration curve, the planning and performance of phase I trials cannot be based on animal experiments only, but should also be based on in vitro data on human tissues (EORTC Pharmacokinetics and Metabolism Group, 1987). The toxicity of new substances against human haemopoietic tissue has been assessed in clonogenic tests on bone marrow cells after exposure to the drug under study. A number of agents were tested by this method, which proved to predict myelotoxicity reliably for many substances (Du et al, 1990, 1991). These tests can also predict the rate of increase in myelotoxicity during dose escalation as well as the interpatient variability (Parchment et al, 1993).

We decided to use a similar type of test to study DNA minor groove alkylators, a family of drugs for which phase I/II evaluations have recently been started. These drugs appear to display a high DNA sequence specificity of alkylation (Broggini et al, 1991; D'Incalci, 1994), which in turn could lead to a higher specificity of

**Figure 1** Chemical structure of the distamycin derivatives used

*Received 31 July 1996*
*Revised 30 September 1996*
*Accepted 7 October 1996*

*Correspondence to:* M Ghielmini the anti-tumour effect. Members of this family are carzelesin, which is a CC-1065 derivative, and the distamycin derivatives, some of which have been shown to possess anti-tumour activity in several models (Pezzoni et al, 1991; Li et al, 1992; D'Alessio et al, 1994). In order to compare the in vitro activity of these substances, we determined their cytotoxicity in tumour cell lines and their toxic effects on human haemopoietic tissue. The latter are usually studied on human bone marrow cells, but these are difficult to obtain in large amounts. Given the similarity between bone marrow (BM) and human cord blood-derived haematopoietic cells (Hows et al, 1992), we developed a model based on cord blood (hCB) cells and used it, after validation, to compare the myelotoxicity of carzelesin and of four distamycin derivatives.

### MATERIALS AND METHODS

### Cytotoxic treatment of haemopoietic cells

The distamycin derivatives tallimustine (FCE24517), FCE25450, FCE28164 and FCE28102 (Figure 1) were provided by Pharmacia (Milan, Italy), and carzelesin was a gift from Upjohn (Kalamazoo, MI, USA). The classical alkylating agent melphalan was used as a reference compound. All drugs were provided as dry powder and were reconstituted with dimethylsulphoxide (DMSO) (distamycin analogues), hydrochloric acid (L-PAM) or *N*,*N*,dimethylacetamide (carzelesin) and diluted with medium. To avoid degradation of the drugs, all solutions were prepared freshly before each experiment. The actual concentration of the solutions was verified by light absorption on a standard densitometer.

BM cells were obtained by aspiration of the posterior iliac crests of seven normal volunteers (aged 32–45). hCB was obtained from placentas after vaginal deliveries. Cells from 14 hCB samples were used. In all cases, the samples were collected in sterile, heparin-containing tubes, stored at 4°C and processed within 48 h. Cells were separated by density centrifugation on a Ficoll gradient to obtain mononuclear cells (MNCs), which were then adherent cell depleted by overnight incubation in 20% fetal calf serum (FCS) in Iscove's modified Dulbecco medium (IMDM) in plastic flasks. Aliquoted cells were cryopreserved at –80°C in 10% DMSO until use.

After thawing, cells were exposed for 1 h to the drug to be tested at 37°C in IMDM. Cells were then washed twice with medium before being plated on clonogenic assays. The experiments with tallimustine were performed on both BM and hCB, and the other drugs were tested on hCB cells only. Tallimustine was also tested in continuous exposure, by plating BM cells with different concentrations of the drug, which then remained in the medium for the subsequent 2 weeks. For each drug, a first set of 3–6 experiments was set up at concentrations ranging over four logs, while in a second set of 3–6 experiments doses were chosen within the $ID_{30}$–$ID_{90}$ range to fine tune the dose–response curve at the critical concentrations.

### Clonogenic assays

Clonogenic assays were performed according to previously described methods (Coutinho et al, 1993). Briefly, cells were plated in methylcellulose and IMDM, supplemented with 30% FCS, 1% bovine serum albumin (BSA), 10% conditioned medium from the bladder carcinoma cell line 5637 and 2 IU of recombinant erythropoietin. Triplicate cultures were prepared with a final concentration of 1 or 2 × $10^5$ MNC $ml^{-1}$ in 4-well tissue culture plates (Falcon). The plates were incubated for 14 days at 37°C in 5% carbon dioxide and fully humidified air. Colonies were scored on an inverted microscope at 7 days (GM-CFCd7 and CFU-E) and at 14 days (GM-CFCd14, BFU-E and Mix-CFC) according to established criteria: GM-CFC contain at least 50 cells, while a CFC-E is a group of at least five haemoglobinized cells. BFU-E are composed of at least three clusters of haemoglobinized cells near to each other. Mix-CFC are colonies containing both myeloid and erythroid components (Coutinho et al, 1993; Lewis et al, 1994).

### Cell line experiments

Monolayer cultures of human colon adenocarcinoma LoVo (Drewinko et al, 1976) and HT-29 (Fogh and Trempe, 1975) were maintained in Ham's F12 medium supplemented with 1% vitamins (Vitamins BME solution), 2mM L-glutamine and 10% FCS. The human lymphoblastic leukaemia cell line CEM (Beck et al, 1979; Danks et al, 1987) was maintained in minimal essential medium (MEM) supplemented with 10% inactivated FCS and 1% vitamin, and the chronic myeloid leukaemia EM-2 (Keating, 1987) and T-cell leukaemia Jurkatt (Weiss et al 1985) human cell lines were grown in RPMI supplemented with 10% FCS, 2 mM L-glutamine, penicillin and streptomycin.

The drug concentrations required for 70% cell growth inhibition ($IC_{70}$) were determined for LoVo and HT-29 cells using a single-cell plating technique. Exponentially growing cells were seeded 48 h before treatment and then exposed to drugs for 1 h. The medium was removed and cells were incubated in drug-free medium. The number of adherent colonies (at least 50 cells) was determined by manual counting on a light microscope after 8–10 days of incubation at 37°C in 5% carbon dioxide. In vitro drug sensitivity against EM2, Jurkatt and CEM cells was evaluated by counting surviving cells. Exponentially growing cells were seeded in test tubes (1×$10^5$ cells $ml^{-1}$, 2 ml per tube) in the presence of various concentrations of drug. The incubation mixture was kept at 37°C for 1 h, and cells were then washed and incubated for 48 h in drug-free medium. The inhibition of cell growth was evaluated by counting surviving cells with a Coulter counter.

The antiproliferative activity of the drugs was calculated from dose–response curves and expressed as $IC_{70}$ (dose causing the inhibition of cell growth in treated cultures relative to untreated controls).

**Figure 2** Dose–response curve of hCB cells on GM-CFCd14 exposed for 1 h to tallimustine. Median of seven experiments (——) and range (• • •). $ID_{70}$, concentration at which 30% of colonies survive

**Figure 3** Dose–response curve of BM and hCB cells on GM-CFCd14 exposed for 1 h to tallimustine. Median of seven experiments on hCB (——) and of seven experiments on BM (• • •). Error bars represent range values

**Table 1** Median $ID_{70}$ and range (ng $ml^{-1}$) of minor groove alkylators and L-PAM on hCB cells after 1 h exposure

| | GM-CFCd7 | GM-CFCd14 | CFC-E | BFU-E |
|---|---|---|---|---|
| Tallimustine | 145 | 165 | 180 | 220 |
| (*n* = 8) | (90–225) | (110–235) | (90–250) | (175–310) |
| FCE25450 | 410 | 630 | 390 | 460 |
| (*n* = 8) | (370–540) | (420–810) | (330–460) | (410–690) |
| FCE28102 | 2600 | 7700 | 2200 | 4600 |
| (*n* = 6) | (800–4500) | (3200–9300) | (800–4500) | (1900–8700) |
| FCE28164 | 310 | 420 | 570 | 790 |
| (*n* = 7) | (100–470) | (190–470) | (150–700) | (610–870) |
| Carzelesin | 1.9[a] | 3.6[a] | 2.2 | 1.9 |
| (*n* = 11) | (1.7–5) | (2.2–6.3) | (<1–3.6) | (1.2–4.7) |
| L-PAM | 640[a] | 1420[a] | 600[b] | 860[b] |
| (*n* = 6) | (590–760) | (1040–2010) | (260–780) | (620–980) |

[a]GM-CFC d7 vs GM-CFC d14, $P<0.05$. [b]CFC-E vs BFU-E, $P<0.05$.

**Table 2** Rate of increase in myelotoxicity of distamycin derivatives, carzelesin and L-PAM (1 h exposure)

| | GM-CFCd7 | GM-CFCd14 | CFC-E | BFU-E |
|---|---|---|---|---|
| Tallimustine | 30 | 38 | 25 | 52 |
| FCE25450 | 47 | 43 | 120 | 57 |
| FCE28102 | 15 | 11 | 21 | 10 |
| FCE28164 | 12 | 48 | 26 | 50 |
| Carzelesin | 40 | 30 | 13 | 48 |
| L-PAM | 20 | 27 | 36 | 80 |

### Analysis of data and statistics

Dose–response curves were produced by computer using a standard software program (Excel version 5.0). The concentration inhibiting the growth of 70% of CFC ($ID_{70}$) was extrapolated from the curves (Figure 2), and the $ID_{70}$ on GM-CFC d14 was taken as an index of myelotoxicity. For each drug and progenitor type, the slope of the dose–response curve was also calculated. This parameter, which others have called 'rate of increase in myelotoxicity' (Parchment et al, 1993), was determined by measuring the percentage increase in cell inhibition when doubling the drug dose in the interval between $ID_{30}$ and $ID_{80}$, a dose range which included the linear part of the curve for all substances. The ratio of the average $IC_{70}$ of the drugs against five tumour cell lines was used as an anti-tumour index. An 'in vitro therapeutic index' could then be calculated by dividing the myelotoxicity index ($ID_{70}$) by the anti-tumour index ($IC_{70}$).

For each drug, the analysis of differences in the cell inhibition of the various progenitor cells was performed using parametric and non-parametric one-way analysis of variance. Whenever a statistically significant result for the global *F*-test was detected, multiple pairwise comparisons were performed. The adjustments of *P*-values for multiple comparisons were obtained using the Bootstrap regression model (Friedman, 1981).

### RESULTS

Initial studies were performed on BM cells to evaluate the concentration–cytotoxicity curve of 1 h exposure to tallimustine in a range of drug concentrations between 100 and 400 ng $ml^{-1}$. Linearity was observed in the range between 20 and 200 ng $ml^{-1}$ (Figure 3). Subsequent experiments were set up with tallimustine on both BM and hCB cells at doses ranging from 20 to 200 ng $ml^{-1}$, constituting the linear part of the curve. Figure 3 shows a comparison between the toxicity of tallimustine (1 h exposure) for BM and hCB cells on GM-CFCd14. Even though a remarkable variability was observed, similar sensitivity to the drug was observed for both cell sources, the median $ID_{70}$ for GM-CFCd14 being 135 ng $ml^{-1}$ (range 105–240 ng $ml^{-1}$) for BM and 165 ng $ml^{-1}$ (110–235 ng $ml^{-1}$) for hCB.

We then tested, on hCB only, the effect of 1 h exposure to the three other distamycin derivatives, carzelesin and L-PAM. Cell inhibition was determined for all progenitors assessable by this assay, i.e. GM-CFCd7 and d14, CFU-E, BFU-E and CFU-mix. For all the types of cells, the $ID_{70}$ was calculated, except for CFU-mix, which were found in low numbers (even in controls) and hence did not allow any analysis. Table 1 reports the median $ID_{70}$ of each drug against any progenitor type. Overall, all drugs appear to be more toxic against more differentiated progenitors (CFCd7) than against more immature progenitors (CFCd14). The differences observed, however, were not statistically significant, with the exception of carzelesin and L-PAM. Carzelesin was the most potent drug. Tallimustine was the most potent among distamycin analogues, while L-PAM had an intermediate potency between FCE25450 and FCE28102.

The cytotoxicity of different exposure times was studied only for tallimustine. The $ID_{70}$ for GM-CFCd7 from hCB was 130 ng $ml^{-1}$ (range 110–240 ng $ml^{-1}$) for 1 h exposure and 13 ng $ml^{-1}$ (4–28 ng $ml^{-1}$) for continuous exposure; for GM-CFCd14, the corresponding figures were 135 ng $ml^{-1}$ (range 105–240 ng $ml^{-1}$) and 16 ng $ml^{-1}$ (2–32 ng $ml^{-1}$) respectively. It appears that, for this drug, continuous exposure is approximately ten times more myelotoxic than a 1-h exposure, suggesting that the myelotoxicity is schedule dependent.

Table 2 reports the rate of increase in myelotoxicity for each drug and cell type. For GM-CFCd14, tallimustine, FCE28164 and FCE25450 showed the highest rate of increase, corresponding to the steepest curve. FCE28102 had the lower rate of increase. This order did not apply to the other precursors.

The cytotoxic activity of a 1-h treatment with distamycin derivatives, carzelesin and L-PAM against five tumour cell lines is

© Cancer Research Campaign 1997

**Table 3** Cytotoxicity of minor groove alkylators and L-PAM on tumour cell lines: $IC_{70}$ ± s.e. (ng $ml^{-1}$) after 1 h exposure

| Compound | EM 2 | Jurkatt | CEM | HT-29 | LoVo | Mean |
|---|---|---|---|---|---|---|
| Tallimustine | 152 ± 25 | 49 ± 11 | 47 ± 9 | 328 ± 75 | 245 ± 33 | 165 |
| FCE25450 | 157 ± 26 | 48 ± 15 | 95 ± 22 | 624 ± 24 | 97 ± 4 | 204 |
| FCE28102 | 1891 ± 293 | 319 ± 67 | 1770 ± 116 | 16096 ± 2081 | 3958 ± 585 | 4807 |
| FCE28164 | 312 ± 11 | 50 ± 12 | 69 ± 12 | 712 ± 103 | 376 ± 39 | 304 |
| L-PAM | 2770 ± 95 | 1763 ± 48 | 1287 ± 341 | 9020 ± 765 | 5023 ± 177 | 3954 |
| Carzelesin | 0.079 ± 0.01 | 0.016 ± 0.001 | 0.26 ± 0.01 | 0.64 ± 0.02 | 0.16 ± 0.015 | 0.23 |

EM2, human chronic myeloid leukaemia; Jurkatt, human T-cell leukaemia; CEM, human lymphoblastic leukaemia; HT-29 and LoVo, human colon adenocarcinoma.

**Table 4** Myelotoxic and anti-tumour effect of drugs

| | L-PAM | Tallimustine | FCE25450 | FCE28102 | FCE28164 | Carzelesin |
|---|---|---|---|---|---|---|
| Myelotoxic dose ($ID_{70}$ on GM-CFC d14 from hCB) | 1420 | 165 | 630 | 7700 | 420 | 3.6 |
| Cytotoxic dose ($IC_{70}$ on cancer cell lines) | 3954 | 165 | 204 | 4807 | 304 | 0.23 |
| In vitro therapeutic index | 0.4 | 1 | 3.1 | 1.6 | 1.4 | 16 |

In vitro therapeutic index: $ID_{70}$ on hCB/$IC_{70}$ on cancer cell lines.

reported in Table 3. Acute leukaemia cell lines were the most sensitive, while the response of the other cell lines was more variable. Table 4 reports the in vitro therapeutic index of each drug. It appears that carzelesin, the most potent compound, has the best in vitro therapeutic index. In fact, the dose of carzelesin that is toxic against tumour cell lines is approximately 16 times lower than the one that is toxic against human haematopoietic cells or, in other words, the cytotoxic dose is 16 times lower than the myelotoxic one. For the other compounds, differences between cytotoxic and myelotoxic doses are not so strikingly different.

### DISCUSSION

To gain more insight into the characteristics of minor groove alkylators and to optimize the planning of phase I trials with these drugs, we performed myelotoxicity experiments on human haematopoietic tissue, using cord blood as a source of cells. The advantages of this cell source for pharmacological testing are that they are easily obtained and available in abundance. Haematopoietic stem and precursor cells are normally present in fetal blood and were shown to share many features with BM cells (Hows et al, 1992), although some immunophenotypic and in vitro growth characteristics are not completely superimposable (Hao et al, 1995; Fritsch et al, 1996). Recently, Léglise et al (1996) demonstrated the equivalence of both stem cell sources for in vitro myelotoxicity tests for several classes of drugs, including anticancer, antibiotic and antiviral drugs.

Experiments performed in our laboratory with doxorubicin and other anthracyclines produced very similar results on hCB and on BM (manuscript in preparation). The equivalence of BM and hCB for pharmacology tests is also supported by our results with tallimustine, showing similar median $ID_{70}$ for the two sources, i.e. 135 ng $ml^{-1}$ (range 105–240 ng $ml^{-1}$) and 165 ng $ml^{-1}$ (range 110–235 ng $ml^{-1}$) respectively. In addition, the $ID_{70}$ we obtained with L-PAM for GM-CFC and BFU-E on hCB cells were similar to those reported by other groups on BM cells (Du et al, 1990). Our results are not in agreement with those of Volpe et al (1992), who tested tallimustine on BM cells; in their hands, tallimustine was shown to be more toxic than in ours, and erythroid cells appeared to be more sensitive than myeloid progenitors. These discrepancies might not be related to the cell source but to technical differences, such as mode of drug dilution, time of exposure (4 h instead of 1 h), smaller number of experiments and different range of concentrations tested. We have observed an important variability of $ID_{70}$ on all the progenitors tested because cells from some sources are intrinsically more sensitive to drugs than others. This corresponds to the difference in chemotherapy tolerance seen among patients in the clinic, the cause of which is still mostly unknown, even though differences in intracellular distribution, active cell excretion and integrity of repair mechanisms can be speculated.

To ensure clinically relevant information, we studied the dose–response curve in the range of clinically relevant concentrations ($ID_{30}$–$ID_{80}$). For tallimustine, for example, the $ID_{30}$ is around 100 ng $ml^{-1}$ and the $ID_{90}$ is around 300 ng $ml^{-1}$, so that the testing of concentrations much below or above this range would not yield much information. The estimation of the toxic drug levels could become very helpful in the setting of pharmacologically guided dose escalation, whereby the goal would become the attainment of plasma concentrations similar to those achieved in animals at toxic doses and within the range of those found to be cytotoxic in clonogenic tests (Du et al, 1995). The drug concentrations defined in our experiments are in agreement with those achieved in humans: the $ID_{70}$ range found for tallimustine against myeloid progenitors (90–235 ng $ml^{-1}$) is in the range (104–189 ng $ml^{-1}$) of those reported in four patients 1 h after administration of doses near the MTD (Sessa et al, 1994). A similar trend was also seen for carzelesin (in vitro 1.7–6.3 ng $ml^{-1}$, in humans 1–2 ng $ml^{-1}$) (Wolff et al, 1996) and L-PAM (in vitro 590–2100 ng $ml^{-1}$, in humans 100–800 ng $ml^{-1}$ at a dose below the MTD) (Zucchetti et al, 1988). A similar in vivo–in vitro correlation for myelotoxicity between concentrations active on clonogenic tests and plasma concentrations achieved in phase I studies was found with other compounds such as pyrazolacridine (Parchment et al, 1994; Rowinsky et al, 1995).

© Cancer Research Campaign 1997

Clonogenic tests could also help to define which component of the drug exposure (peak, AUC, time above a threshold concentration) is the most important for toxicity. For tallimustine, all progenitors were killed when exposed in vitro for 1 h at 400 ng $ml^{-1}$ or higher. Based on our data showing that a continuous exposure of 40 ng $ml^{-1}$ is as toxic as 400 ng $ml^{-1}$ for 1 h, we believe that AUC and not the time above a critical concentration is the main determinant of toxicity.

The pattern of toxicity of a drug on haemopoietic cells in vitro could be useful for predicting the pattern of myelotoxicity in humans. In the process of blood cell differentiation, a common stem cell capable of autoreplication gives rise to a lymphatic and a myeloid precursor. The latter, which corresponds in vitro to the long-term culture-initiating cell (LTC-IC), generates progenitor cells of granulocytes and macrophages (GM-CFCd14), erythrocytes (BFU-E) and megakaryocytes (BFU-Meg), which are clonogenic and hence recognizable in vitro. These progenitors then differentiate into the final mature blood elements through an intermediate step of cells (Lewis et al, 1994) which are still clonogenic (GM-CFCd7, CFC-E and Meg-CFC). Our assay can test myeloid and erythroid clonogenic cells, while the analysis of LTC-IC requires more sophisticated and time-consuming methods based on the long-term culture principle (Hows et al, 1992). The study of megakaryocyte progenitors would also be relevant for the clinic, but this technique is still insufficiently standardized to be applied.

From our results, we could predict that tallimustine and FCE28102 (which seemed to be more toxic against the myeloid than the erythroid line) would induce, mainly, a selective granulocytopenia, while L-PAM, carzelesin and the other distamycin derivatives would mainly affect the erythroid line. Preliminary clinical data with tallimustine and carzelesin support this hypothesis. Even though anaemia is clinically less relevant and more difficult to evaluate than leucopenia, the difference in the toxic effects on the myeloid and erythroid lines is theoretically interesting as it illustrates how changes in the chemical structure of the alkylating moiety can affect the response of each cell type.

It should be pointed out that the mechanism of cytotoxicity of minor groove binders has still not been fully elucidated. The compounds that have been investigated in greater detail are CC-1065 or its analogues, including carzelesin and the distamycin derivative tallimustine. CC-1065 and its analogues alkylate adenine N3 with a high selectivity for adenine located in the sequence AAAAA or PUNAAA, whereas tallimustine alkylates adenine N3 located in the hexamer TTTTGA. Therefore, even though all these compounds can be classified as DNA minor groove alkylators, it appears that they can hit different DNA sequences, possibly causing function impairment of different genes. Recent studies have in fact shown that both the CC-1065 type of compounds and distamycin derivatives can selectively block the sequence-specific binding of transcription factors, thus impairing the transcription of genes regulated by these proteins. It is then conceivable that different minor groove binders possess different sequence preferences for DNA binding, thereby causing different biological effects, either in terms of their anti-tumour activity or of their toxicity. This could be an explanation for the difference in the pattern of myelotoxicity of tallimustine and carzelesine and of tallimustine and some of the other distamycin derivatives, such as FCE 25450.

For all drugs tested, the less differentiated precursors (GM-CFCd14 and BFU-E) seemed to be less affected than the more mature ones (GM-CFCd7 and CFC-E). The higher resistance to cytostatics could be interpreted as a higher capability of immature cells to repair DNA or, alternatively, be attributed to the smaller proportion of immature cells that are in a proliferative state.

We can hypothesize that similar resistance mechanisms could play a role in the different patterns of myelotoxicity in humans, for example tallimustine was shown to cause an early, brief and selective granulocytopenia (Sessa et al, 1994) while carzelesin induced a delayed, long-lasting and cumulative pancytopenia (Wolff et al, 1996). For tallimustine, we can hypothesize that early precursor cells (i.e. LTC-IC) might be more resistant while clonogenic cells such as GM-CFC are very sensitive to the drug; for carzelesin, the contrary might be true. These issues could be elucidated by the long-term culture system, which could help to define the differentiation step of the haematopoietic process that is most affected by the exposure to a given cytotoxic agent.

Minor-groove alkylators remain a promising field of research. Other distamycin analogues could now enter phase I trials, and data from our clonogenic tests will be useful for selecting the compounds most promising for clinical development. From the results of this study, it appears that the analogue with the best therapeutic index (three times better than tallimustine, approximately nine times better than L-PAM) is the distamycin derivative FCE25450, which bears an $\alpha$-bromoacrylic group, and its further development in phase I trials should be encouraged. On the other hand, the excellent in vitro therapeutic index of carzelesin has not yet been confirmed in clinical studies, whereby delayed, long-lasting and cumulative myelosuppression represents the dose-limiting toxicity.

In conclusion, we have shown that valuable predictive information on the myelotoxicity of cytotoxic agents can be obtained by clonogenic assays on human cord blood cells. By comparing new analogues to known drugs, a therapeutic index as well as the starting dose and the magnitude of dose escalations for clinical studies could be suggested.

### ACKNOWLEDGEMENTS

We are particularly indebted to the midwives of the Ospedale di Monza for their help in collecting fresh cord blood for use in these experiments. We are especially thankful to Dr A Biondi, Monza for fruitful discussions and suggestions. This study was supported by grant no. 32–39764.93 of the Swiss National Research Fund.

### REFERENCES

Beck WT, Muelle TJ and Tanzler LR (1979) Altered surface membrane glycoproteins in vinca-alkaloid-resistant human leukemic lymphoblasts. *Cancer Res* **37**: 5455–5460

Broggini M, Erba E, Ponti M, Ballinari D, Geroni C, Spreafico F and D'Incalci M (1991) Selective DNA interaction of the novel distamycin derivative FCE 24517. *Cancer Res* **51**: 199–204

Collins JM, Grieshaber CK and Chabner BA (1990) Pharmacologically guided phase I clinical trials based upon preclinical drug development. *J Natl Cancer Inst* **82**: 1321–1326

Coutinho LH, Gilleece MH, De Wynter EA, Will A and Testa NG (1993) Clonal and long-term cultures using human bone marrow. In *Haemopoiesis. A Practical Approach*, Testa NG and Molineux G (eds), pp. 75–105. Oxford University Press: New York

D'Alessio R, Geroni C, Biasoli G, Pesenti E, Grandi M and Mongelli N (1994) Structure-activity relationship of novel distamycin A derivatives: synthesis and antitumor activity. *Bioorganic and Medicinal Chemistry Letters* **4**: 1467–1472

D'Incalci M (1994) DNA-minor-groove alkylators, a new class of anticancer agents. *Ann Oncol* **5**: 877–878

© Cancer Research Campaign 1997

Danks MK, Yalowich JC and Beck WT (1987) Atypical multiple drug resistance in a human leukemic cell line selected for resistance to teniposide (VM-26). *Cancer Res* **47**: 1297–1301

Davis LE, Alberts DS, Plezia PM, ROE DJ and Griswold DP (1988) Predictive model for plasma concentration-versus-time profiles of investigational anticancer drugs in patients. *J Natl Cancer Inst* **80**: 815–819

Drewinko B, Romsdahal MM, Jang LJ, Ahearn MI and Trujillo JM (1976) Establishment of a human carcinoembrionic antigen producing colon adenocarcinoma cell line. *Cancer Res* **36**: 467

Du DL, Volpe DA, Grieshaber CK and Murphy MJJ (1990) Effects of L-phenylalanine mustard and L-buthionine sulfoximine on murine and human hematopoietic progenitor cells in vitro. *Cancer Res* **50**: 4038–4043

Du DL, Volpe DA, Grieshaber CK and Murphy MJJ (1991) Comparative toxicity of fostriecin, hepsulfam and pyrazine diazohydroxide to human and murine hematopoietic progenitor cells in vitro. *Invest New Drugs* **9**: 149–157

Du XX, Scott D, Yang ZX, Cooper R, Xiao XL and Williams DA (1995) Interleukin-11 stimulates multilineage progenitors, but not stem cells, in murine and human long-term marrow cultures. *Blood* **86**: 128–134

Eortc Pharmacokinetics and Metabolism Group (1987) Pharmacokinetically guided dose escalation in phase I clinical trials. Commentary and proposed guidelines. *Eur J Cancer Clin Oncol* **23**: 1083–1087

Fogh J and Trempe G (1975) New human tumor cell lines. In *Human Tumor Cell 'In Vitro'*, Fogh J (ed.), p. 115. Plenum Publishing: New York

Friedman DA (1981) Bootstrap regression models. *Ann Stat* **9**: 1218–1228

Fritsch G, Stimpfl M, Kurz M, Printz D, Buchinger P, Fischmeister G, Hoecker P and Gadner H (1996) The composition of CD34 subpopulations differs between bone marrow, blood and cord blood. *Bone Marrow Transplantation* **17**: 169–178

Gianni L, Viganó L, Surbone A, Ballinari D, Casali P, Tarella C, Collins JM and Bonadonna G (1990) Pharmacology and clinical toxicity of 4′-Iodo-4′-deoxydoxorubicin: an example of successful application of pharmacokinetics to dose escalation in phase I trials. *J Natl Cancer Inst* **82**: 469–477

Hao Q-L, Shah AJ, Thiemann FT, Smogarzewska EM and Crooks GM (1995) A functional comparison of CD34+CD38– cells in cord blood and bone marrow. *Blood* **86**: 3745–3753

Hows JM, Bradley BA, Marsh JCW, Luft T, Coutinho LH, Testa NG and Dexter TM (1992) Growth of human umbilical-cord blood in longterm haemopoietic cultures. *Lancet* **340**: 73–75

Keating A (1987) Ph positive CML cell lines. *Clin Hematol* **1**: 1021–1029

Lewis JL, Blackett NM and Gordon MY (1994) The kinetics of colony formation by CFU-GM in vitro. *Br J Haematol* **88**: 440–442

Léglise MC, Darodes DE Tailly P, Vignot JL, Le Bot MA, Le Roux A-M and Riché C (1996) A cellular model for drug interactions on hematopoiesis: the use of human umbilical cord blood progenitors as a model for the study of drug-related myelosuppression of normal hematopoiesis. *Cell Biol Toxicol* **12**: 39–53

Li LH, Dekoning TF, Kelly RC, Krueger WC, Mcgoveren JP, Padbury GE, Petzold GL, Wallace TL, Ouding RJ, Prairie MD and Gebhard I (1992) Cytotoxicity and antitumor activity of carzelesin, a prodrug cyclopropylpyrroloindole analogue. *Cancer Res* **52**: 4904–4913

Parchment RE, Huang M and Erickson-Miller CL (1993) Roles for in vitro myelotoxicity tests in preclinical drug development and clinical trial planning. *Toxicol Pathol* **21**: 241–250

Parchment RE, Lorusso PM, Volpe DA, Erickson-Miller CL, Murphy MJJ and Grieshaber CK (1994) In vivo–in vitro correlation of myelotoxicity of 9-methoxypyrazoloacridine (NSC-366140, PD 115934) to myeloid and erythroid hematopoietic progenitors from human, murine, and canine marrow. *J Natl Cancer Inst* **86**: 273–280

Pezzoni G, Grandi M, Biasoli G, Capolongo L, Ballinari D, Giuliani FC, Barbieri B, Pastori A, Pesenti E, Mongelli E and Spreafico F (1991) Biological profile of FCE 24517, a novel benzoyl mustard analogue of distamycin A. *Br J Cancer* **64**: 1047–1050

Rowinsky EK, Noe DA, Grochow LB, Sartorious SE, Bowling MK, Chen T, Lubejko BG, Kaufmann SH and Donehower RC (1995) Phase I and pharmacologic studies of pyrazoloacridine, a novel DNA intercalating agent, on single-dosing and multiple-dosing schedules. *J Clin Oncol* **13**: 1975–1984

Sessa C, Pagani O, Zurlo MG, DE Jong J, Hofmann C, Lassus M, Marrari P, Strolin Benedetti M and Cavalli F (1994) Phase I study of the novel distamycin derivative tallimustine (FCE 24517). *Ann Oncol* **5**: 901–907

Volpe DA, DU DL, Zurlo MG, Mongelli N and Murphy MJ (1992) Comparative in vitro myelotoxicity of FCE 24517, a distamycin derivative, to human, canine and murine hematopoietic progenitor cells. *Invest New Drugs* **10**: 255–261

Weiss A, Wiskocil RL and Stobo JD (1985) The role of T3 surface molecules in the activation of human T cells: a two-stimulus requirement for IL-2 production reflects events occurring at a pre-translational level. *J Immunol* **133**: 123–128

Wolff I, Bench K, Beijnen J, Bruntsch U, Cavalli F, DE Jong J, Groot Y, Van Tellingen O, Wanders J and Sessa C (1996) Phase I clinical and pharmacokinetic study of carzelesin (U-80244) given on a daily x 5 schedule. *Clin Cancer Res* **2**: 1717–1723

Zucchetti M, D'Incalci M, Willems Y, Cavalli F and Sessa C (1988) Lack of effect of cisplatin on i.v. L-PAM plasma pharmacokinetics in ovarian cancer patients. *Cancer Chemother Pharmacol* **22**: 87–89

© Cancer Research Campaign 1997


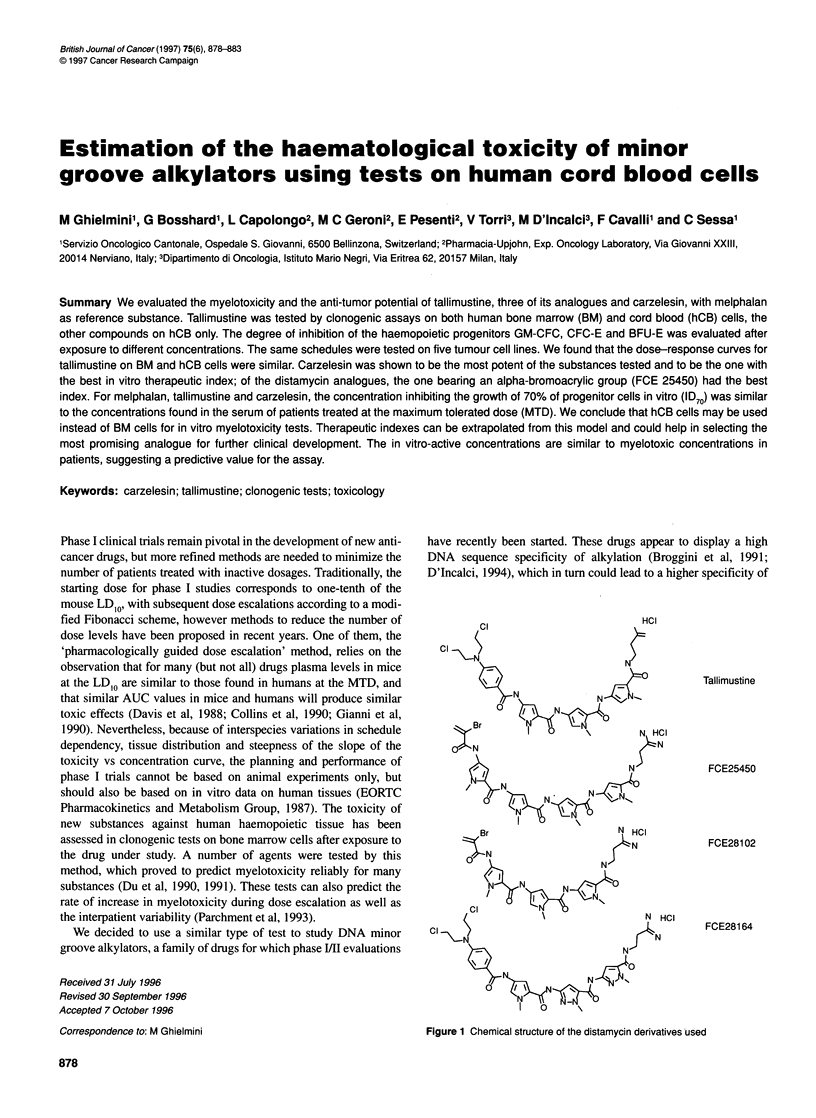

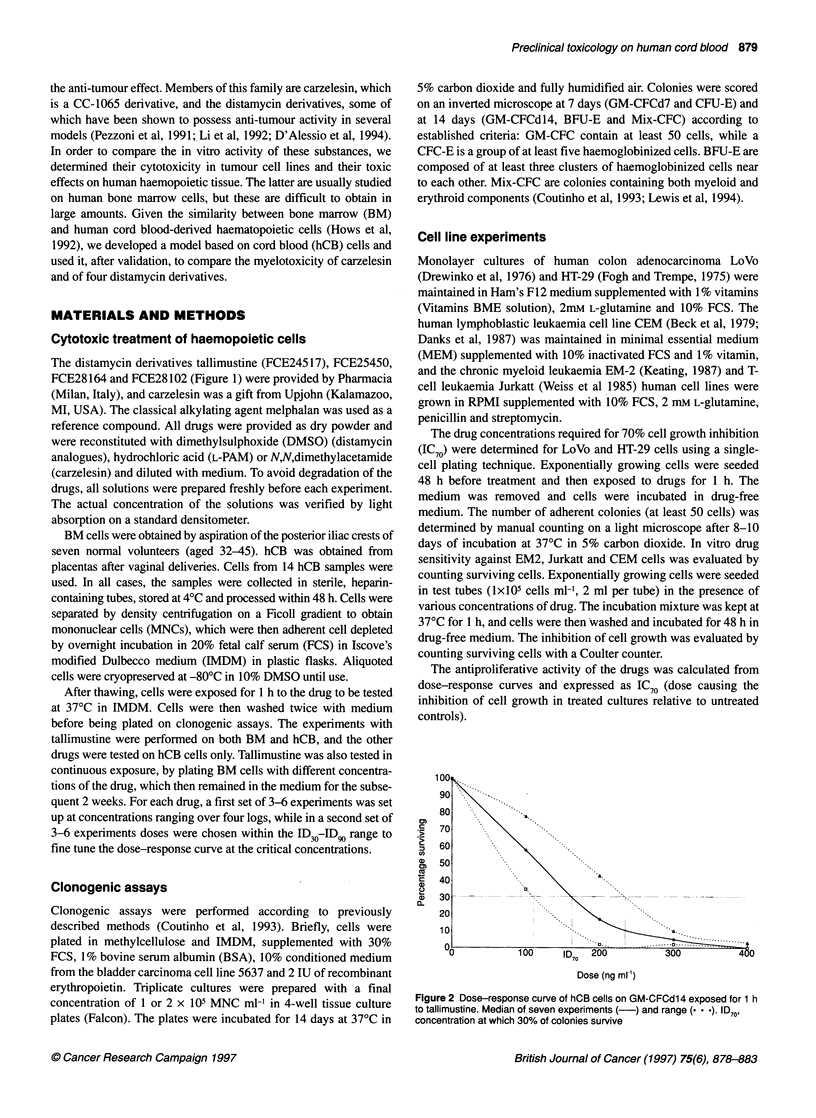

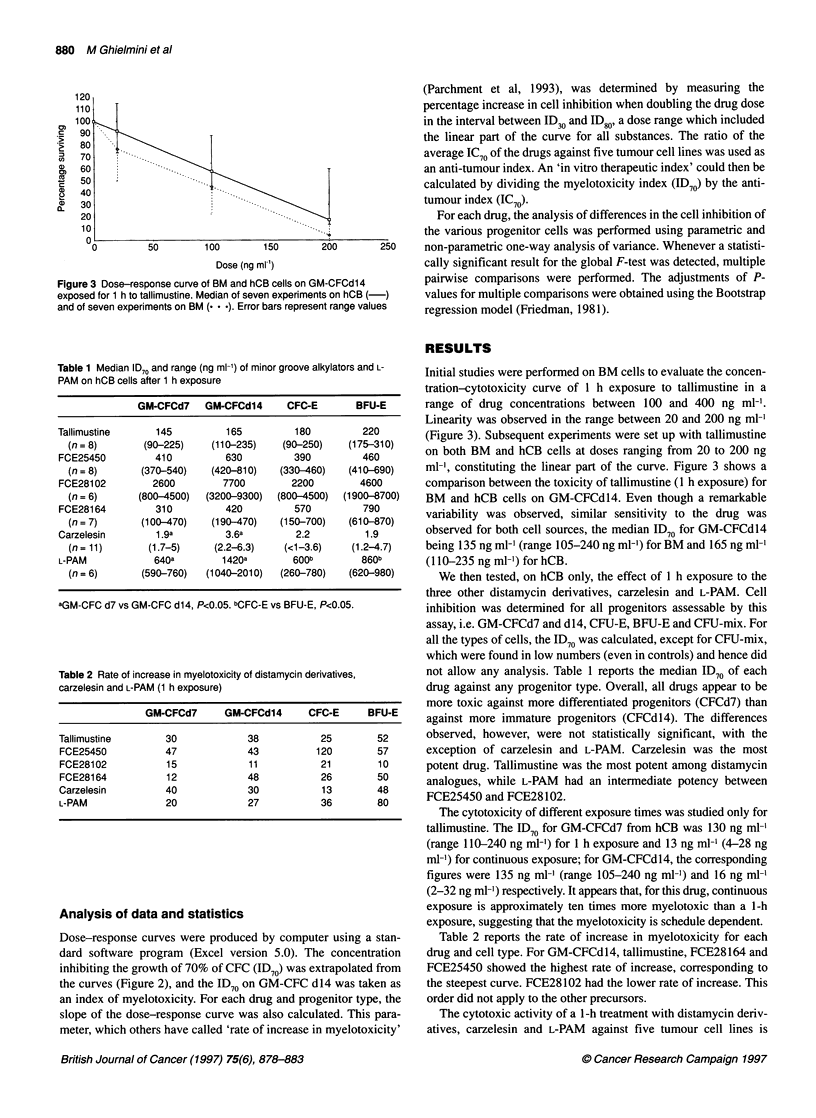

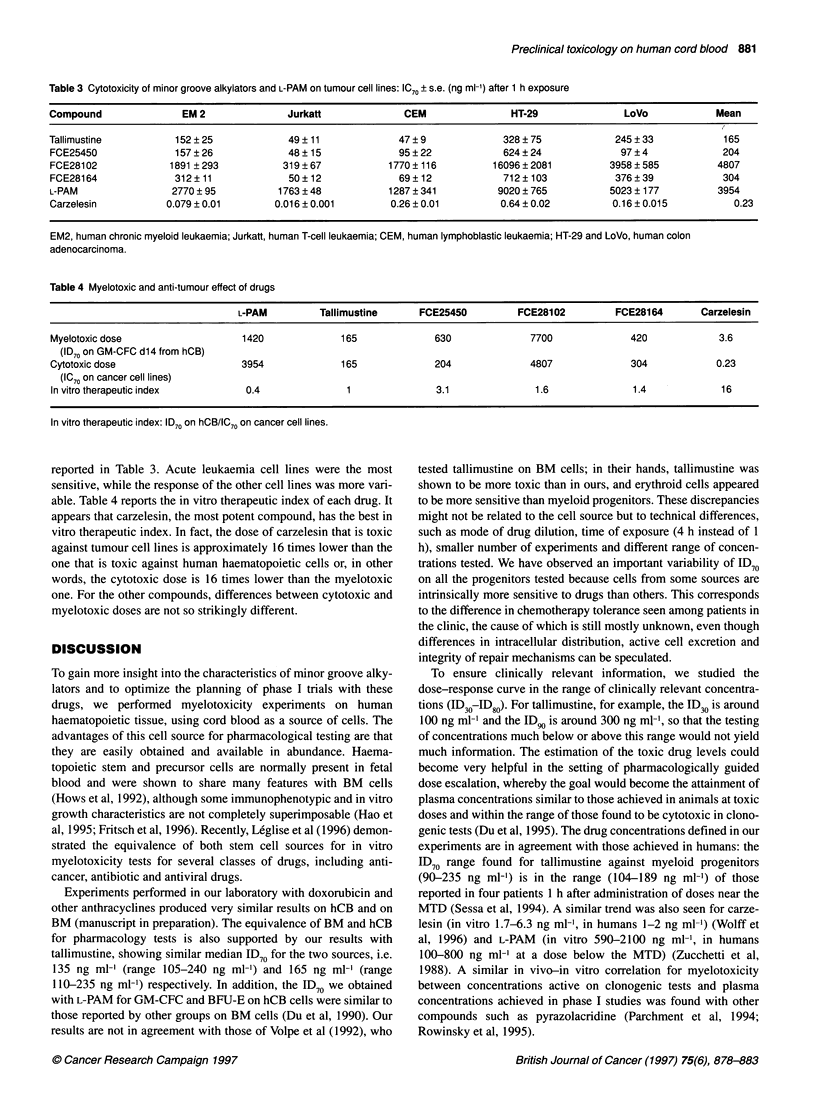

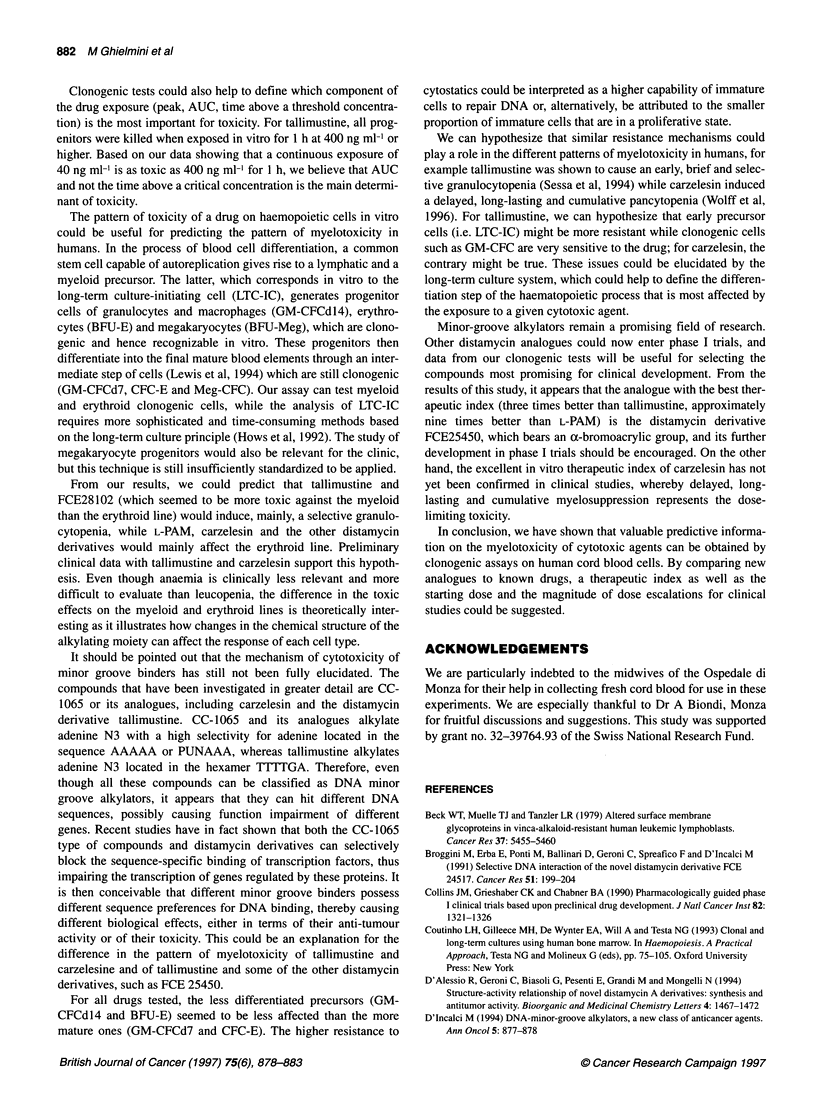

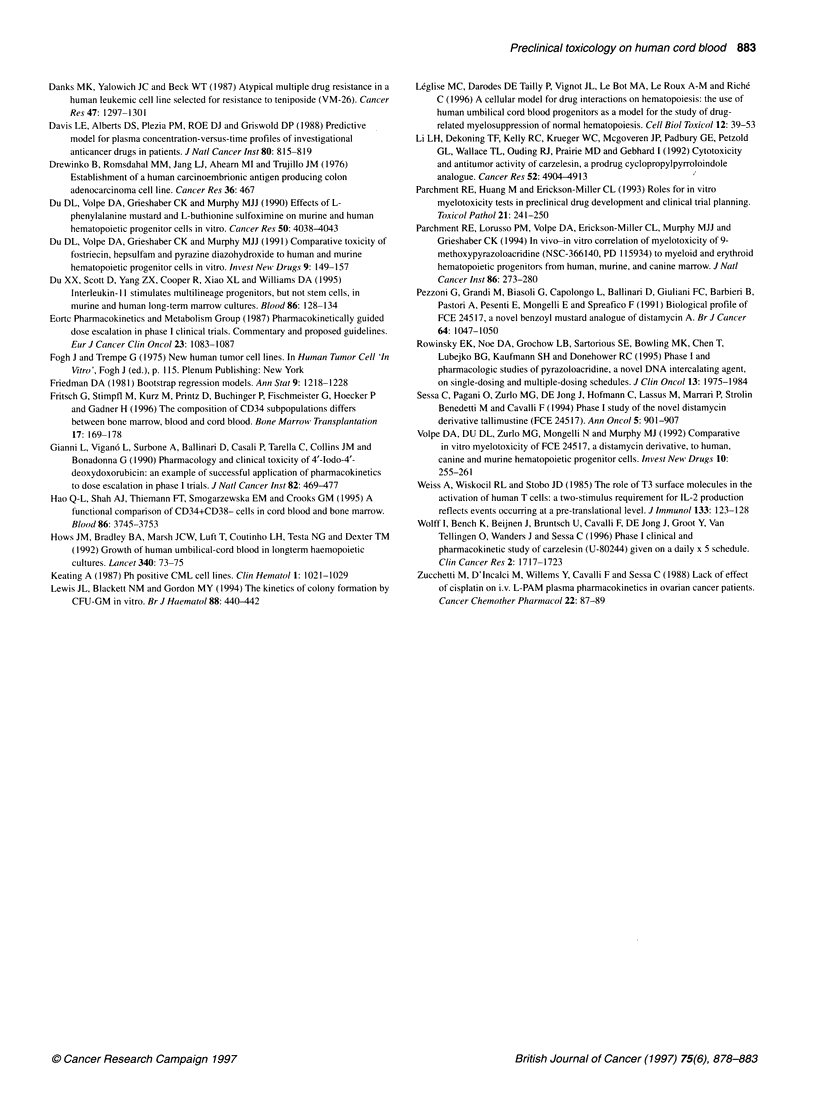


## References

[OCR_00609] Broggini M., Erba E., Ponti M., Ballinari D., Geroni C., Spreafico F., D'Incalci M. (1991). Selective DNA interaction of the novel distamycin derivative FCE 24517.. Cancer Res.

[OCR_00614] Collins J. M., Grieshaber C. K., Chabner B. A. (1990). Pharmacologically guided phase I clinical trials based upon preclinical drug development.. J Natl Cancer Inst.

[OCR_00629] D'Incalci M. (1994). DNA-minor-groove alkylators, a new class of anticancer agents.. Ann Oncol.

[OCR_00637] Danks M. K., Yalowich J. C., Beck W. T. (1987). Atypical multiple drug resistance in a human leukemic cell line selected for resistance to teniposide (VM-26).. Cancer Res.

[OCR_00647] Drewinko B., Romsdahl M. M., Yang L. Y., Ahearn M. J., Trujillo J. M. (1976). Establishment of a human carcinoembryonic antigen-producing colon adenocarcinoma cell line.. Cancer Res.

[OCR_00657] Du D. L., Volpe D. A., Grieshaber C. K., Murphy M. J. (1991). Comparative toxicity of fostriecin, hepsulfam and pyrazine diazohydroxide to human and murine hematopoietic progenitor cells in vitro.. Invest New Drugs.

[OCR_00652] Du D. L., Volpe D. A., Grieshaber C. K., Murphy M. J. (1990). Effects of L-phenylalanine mustard and L-buthionine sulfoximine on murine and human hematopoietic progenitor cells in vitro.. Cancer Res.

[OCR_00661] Du X. X., Scott D., Yang Z. X., Cooper R., Xiao X. L., Williams D. A. (1995). Interleukin-11 stimulates multilineage progenitors, but not stem cells, in murine and human long-term marrow cultures.. Blood.

[OCR_00677] Fritsch G., Stimpfl M., Kurz M., Printz D., Buchinger P., Fischmeister G., Hoecker P., Gadner H. (1996). The composition of CD34 subpopulations differs between bone marrow, blood and cord blood.. Bone Marrow Transplant.

[OCR_00686] Gianni L., Viganò L., Surbone A., Ballinari D., Casali P., Tarella C., Collins J. M., Bonadonna G. (1990). Pharmacology and clinical toxicity of 4'-iodo-4'-deoxydoxorubicin: an example of successful application of pharmacokinetics to dose escalation in phase I trials.. J Natl Cancer Inst.

[OCR_00691] Hao Q. L., Shah A. J., Thiemann F. T., Smogorzewska E. M., Crooks G. M. (1995). A functional comparison of CD34 + CD38- cells in cord blood and bone marrow.. Blood.

[OCR_00696] Hows J. M., Bradley B. A., Marsh J. C., Luft T., Coutinho L., Testa N. G., Dexter T. M. (1992). Growth of human umbilical-cord blood in longterm haemopoietic cultures.. Lancet.

[OCR_00701] Keating A. (1987). Ph positive CML cell lines.. Baillieres Clin Haematol.

[OCR_00703] Lewis J. L., Blackett N. M., Gordon M. Y. (1994). The kinetics of colony formation by CFU-GM in vitro.. Br J Haematol.

[OCR_00713] Li L. H., DeKoning T. F., Kelly R. C., Krueger W. C., McGovren J. P., Padbury G. E., Petzold G. L., Wallace T. L., Ouding R. J., Prairie M. D. (1992). Cytotoxicity and antitumor activity of carzelesin, a prodrug cyclopropylpyrroloindole analogue.. Cancer Res.

[OCR_00707] Léglise M. C., Darodes de Tailly P., Vignot J. L., Le Bot M. A., Le Roux A. M., Riché C. (1996). A cellular model for drug interactions on hematopoiesis: the use of human umbilical cord blood progenitors as a model for the study of drug-related myelosuppression of normal hematopoiesis.. Cell Biol Toxicol.

[OCR_00719] Parchment R. E., Huang M., Erickson-Miller C. L. (1993). Roles for in vitro myelotoxicity tests in preclinical drug development and clinical trial planning.. Toxicol Pathol.

[OCR_00724] Parchment R. E., Volpe D. A., LoRusso P. M., Erickson-Miller C. L., Murphy M. J., Grieshaber C. K. (1994). In vivo-in vitro correlation of myelotoxicity of 9-methoxypyrazoloacridine (NSC-366140, PD115934) to myeloid and erythroid hematopoietic progenitors from human, murine, and canine marrow.. J Natl Cancer Inst.

[OCR_00732] Pezzoni G., Grandi M., Biasoli G., Capolongo L., Ballinari D., Giuliani F. C., Barbieri B., Pastori A., Pesenti E., Mongelli N. (1991). Biological profile of FCE 24517, a novel benzoyl mustard analogue of distamycin A.. Br J Cancer.

[OCR_00738] Rowinsky E. K., Noe D. A., Grochow L. B., Sartorious S. E., Bowling M. K., Chen T. L., Lubejko B. G., Kaufmann S. H., Donehower R. C. (1995). Phase I and pharmacologic studies of pyrazoloacridine, a novel DNA intercalating agent, on single-dosing and multiple-dosing schedules.. J Clin Oncol.

[OCR_00744] Sessa C., Pagani O., Zurlo M. G., de Jong J., Hofmann C., Lassus M., Marrari P., Strolin Benedetti M., Cavalli F. (1994). Phase I study of the novel distamycin derivative tallimustine (FCE 24517).. Ann Oncol.

[OCR_00749] Volpe D. A., Du D. L., Zurlo M. G., Mongelli N., Murphy M. J. (1992). Comparative in vitro myelotoxicity of FCE 24517, a distamycin derivative, to human, canine and murine hematopoietic progenitor cells.. Invest New Drugs.

[OCR_00755] Weiss A., Wiskocil R. L., Stobo J. D. (1984). The role of T3 surface molecules in the activation of human T cells: a two-stimulus requirement for IL 2 production reflects events occurring at a pre-translational level.. J Immunol.

[OCR_00767] Zucchetti M., D'Incalci M., Willems Y., Cavalli F., Sessa C. (1988). Lack of effect of cisplatin on i.v. L-PAM plasma pharmacokinetics in ovarian cancer patients.. Cancer Chemother Pharmacol.

